# Comparative Study of the Physicochemical Properties and Nutritional Quality of Argan Oil from Endemic and Planted *Argania spinosa* (L.) Fruit Trees in South-West Algeria

**DOI:** 10.3390/foods15142557

**Published:** 2026-07-20

**Authors:** Mohammed Ould Safi, Giuseppa Di Bella, Vincenzo Nava, Ambrogina Albergamo, Abdelaali Bencheikh, Said Boudeffeur, Mohammed Boughalia, Réda Kechairi, Angela Giorgia Potortì

**Affiliations:** 1 National Institute of Forestry Research, Baïnem Forest, P.O. Box 37, Chéraga, Algiers 16014, Algeria; moh.safi.inrf@gmail.com; 2 Department of Biomedical, Dental, Morphological and Functional Images Sciences (BIOMORF), University of Messina, Viale Annunziata, 98100 Messina, Italy; gdibella@unime.it (G.D.B.); vnava@unime.it (V.N.); agpotorti@unime.it (A.G.P.); 3 Laboratory of Saharan Natural Resources, Faculty of Natural and Life Sciences, University Ahmed Draia, Adrar 01000, Algeria; bencheikh.aali@univ-adrar.edu.dz; 4 National Institute for Agricultural Research, Street the Brothers OUADEK-BP N 200 Hassen Badi -16200-EL-Harrach, Algiers P5C3+W3, Algeria; saidboudeffeur@yahoo.fr; 5 General Directorate of Forests of Algeria, Road Doudou Mokhtar, Algiers P.O. Box 232, Algeria; medboughalia@gmail.com; 6 Laboratory of Ecology and Management of Natural Ecosystems, Department of Ecology and Environment, Snv/Stu Faculty, Abou Bekr Belkaid University, Tlemcen 13000, Algeria; kechairir79@gmail.com

**Keywords:** argan tree, natural forest, domestication, argan oil, physicochemical quality, fatty acids, phytosterols, inorganic elements, tocopherols, total polyphenols, squalene

## Abstract

*Argania spinosa* tree, notoriously famous for its primary product, the argan oil, is a versatile agroforestry tree that grows wild in Algeria and Morocco. However, recent cultivation efforts have been undertaken in Algeria to protect the species and valorize the local productions of argan oil. Hence, a comparative study was conducted on cold-pressed oils from endemic and planted argan trees, located respectively in Tindouf and Adrar provinces of Southwestern Algeria, to evaluate the impact of plantation on argan oil quality. For each area, three samples of argan oil were produced and analyzed by three analytical replicates. According to the Moroccan guidelines, both products were extra-virgin oils due to favorable saponification indices, free acidities, peroxide values and UV exams. Their fatty acid composition and sterol profile were similar, with high levels of unsaturated fatty acids and peculiar phytosterols beneficial to consumer health. However, the cultivation of *A. spinosa* affected the amounts of tocopherols, total polyphenols and carotenes, squalene, and inorganic elements, which were generally lower in the oil from Adrar than in that from Tindouf. These differences may be related to the different pedoclimatic and agronomic contexts of growth and did not affect the quality and marketability of argan oil. Overall, this study provides preliminary evidence of the feasibility of cultivating *A. spinosa* outside its natural biotope in hyper-arid Algerian regions, producing high-quality oil that could play a crucial socio-economic role in the national economy. However, further studies involving multiple cultivation environments and long-term agronomic and ecological assessments are required to better understand the performance of *A. spinosa* under non-native growing conditions.

## 1. Introduction

*Argania spinosa* (L.) Skeels (*Sapotaceae*) is a perennial tree characterized by fleshy berry fruits, which contain a kernel (almond) that is used to produce an ancient and fascinating elixir known as argan oil. The species is endemic to Central and Southwestern Morocco and Western Algeria, and it has adapted well to arid and semi-arid environments, the argan forest being part of a transitional zone between Mediterranean and Saharan climates [1,2]. The tree plays a relevant ecological role since it protects against soil erosion and desertification, preserves biodiversity, and regulates the local climate by reducing the release of CO_2_ in the atmosphere [3,4]. In addition, it represents a vibrant socio-economic resource, since its main products, i.e., oil and wood, provide rural communities with job opportunities and income diversification [5,6].

It is precisely for the significant ecological, cultural, and economic value that, in 1998, the UNESCO (United Nations Educational Scientific and Cultural Organization) designated the largest argan forest in Southwestern Morocco (Arganeraie) as a biosphere reserve, namely an area reconciling the maintenance of biodiversity with its sustainable use, and, in 2014, included all the practices and know-how concerning the argan tree in the List of the Intangible Cultural Heritage of Humanity, further increasing the global interest in the species [7].

Argan oil is a globally prized food and cosmetic ingredient with many healthy virtues, due to its chemical profile rich in unsaturated lipids and antioxidants, such as tocopherols and phytosterols [8]. In fact, the oil is a traditional component of the North African diet, being highly appreciated for its nutraceutical properties and distinctive nutty flavor [8], and is also well renowned in the cosmetic area for moisturizing skin and hair, and for treating acne, stretch marks, skin irritations and signs of aging [9].

The global argan oil market is growing rapidly due to increasing demand from the cosmetic, medicinal, and food industries [10]. Morocco is the main producer and exporter of high-purity argan oil, which is often certified as organic and ethically sourced. Here, *A. spinosa* grows wild mainly in the structured and dense ecosystem of Arganeraie, which covers over 2.5 million ha, thanks to the combined influence of the Atlantic Ocean, high mountains and sea-level desert. Moreover, a well-established market, led by women’s cooperatives and accounting for over 95% of global output, is centered primarily in the Souss-Massa region [11,12]. Nonetheless, Algeria has recently emerged as a competitor, producing high-quality argan oil through environmentally friendly methods [11]. Algerian argan forests, however, are much smaller, more fragmented (~90,000 ha), and exposed to harsher conditions, such as drought and termite attacks [13,14]. In particular, the natural argan groves, which are historically concentrated in the province of Tindouf, have shrunk due to overgrazing and excessive woodcutting, becoming “relic populations” more vulnerable to degradation and fluctuations in fruit yield and quality [2]. Although Algeria considers argan oil a strategic sector, its market is still limited by informal trade practices and the lack of an effective traceability system, which hinders international exports [15,16].

In this scenario, the development of sustainable strategies for the preservation and propagation of argan trees is necessary to boost domestic production and export capacity. The Algerian government has recently launched several key initiatives for protecting and valorizing the argan tree, including planting *A. spinosa* in its natural habitat and in new areas as well, with the aim to preserve the species’ genetic resources, rehabilitate degraded areas and support the development of a national argan oil sector capable of generating economic opportunities [17].

Indeed, the cultivation of fruit trees has already been demonstrated to be a strategic focus to ensure sustainable income generation and biodiversity conservation in Algeria [18]. Accordingly, several scientific attempts to establish the cultivation of argan trees have been documented in the provinces of Mostaganem, Mascara, Tlemcen, Algiers, Bechar and Adrar, with particularly encouraging results in Adrar and Bechar. In fact, the argan tree has been demonstrated to be suitable for integration into agroforestry systems, and its plantation may effectively serve as a powerful tool for effective socio-economic and environmental management [19,20,21]. However, scientific information regarding the quality of oil produced by cultivated trees remains scarce. Therefore, evaluating whether the introduction of *A. spinosa* into new Algerian agroecological contexts affects the physicochemical and nutritional characteristics of argan oil is essential to support both conservation strategies and future industrial development of the sector.

With these premises, the aim of the present study was to evaluate the success of planting *A. spinosa* outside its biotope in Algeria by focusing on its main product, namely the argan oil. To this purpose, argan oils from native specimens in Tindouf and planted trees in Adrar (Southwestern Algeria) were comparatively investigated for their yield, physicochemical quality, and nutritional and compositional traits.

In this context, this study represents the first investigation aimed at comprehensively comparing the quality of Algerian argan oil obtained from native populations and from trees cultivated outside their natural habitat. Unlike most previous studies focusing almost exclusively on certain compositional aspects of (Moroccan) argan oil, this research offers a broader and more in-depth assessment of *A. spinosa* oil—by including the study of not only its physicochemical parameters but also a detailed characterization of lipid, sterol, antioxidant and mineral constituents—and enables a more robust evaluation of the impact of cultivation in new pedoclimatic contexts. This helps to fill an important knowledge gap and also provides a solid scientific basis for the future agro-industrial development of argan cultivation in Algeria.

Hopefully, the plantation of *A. spinosa* in modern orchards may establish the species as a key crop for oil production. This will open up important economic opportunities for Algeria in the face of the growing demand for high-quality argan oil and help to alleviate the pressure on the wild argan forest.

## 2. Materials and Methods

### 2.1. Plant Material

**Figure 1:**
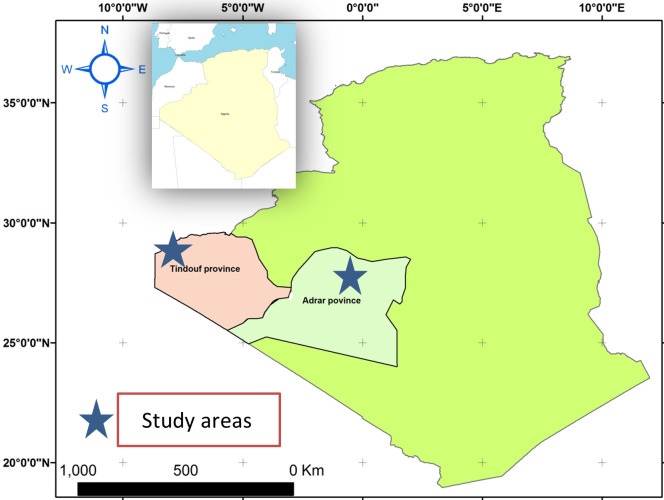
Geographical map of Algeria and its provinces. In the map, the sampling sites of the study, namely Tindouf and Adrar provinces, are also shown.

For this study, native and planted *A. spinosa* trees came respectively from the natural argan grove of the Tindouf province and the station of the National Institute of Forestry Research (NIFR) of Adrar province in Southwestern Algeria (Figure 1). The two sites are approximately 1000 km apart and their geopedoclimatic conditions are reported in Table 1. *A. spinosa* was cultivated using seeds from the natural argan grove in Tindouf. Specifically, the seeds were first planted in the INFR nursery between 2008 and 2009, and n = 87 seedlings were then transplanted to an experimental plot of the INFR station in 2010, as previously described by Ould Safi et al. [13]. The experimental specimens were constantly subjected to a drip irrigation regime that varied based on the age of the plant and season, and they did not receive any chemical inputs. Periodic pruning was also conducted to support their growth and fruit production. At the time of the study, the argan trees were 14 years old and produced ~10 kg of fruit per year.

The sampling of fruits occurred in June 2024. Specifically, n = 500 ripe and healthy fruits (~7 kg) were manually harvested from adult natural trees of unknown age (n = 20) and 14-year-old planted trees (n = 20). The fruits from both sampling sites were transported to the Adrar station (southwestern Algeria) of the National Institute of Forestry Research of Algeria and stored in a dark and dry place, at a constant room temperature, until oil extraction. Additionally, some fruits were authenticated at the University of Tlemecen (northwestern Algeria), with the natural and planted voucher specimens (AS-W 079.24 and AS-D 080.24) being deposited in the university herbarium.

### 2.2. Oil Extraction

The extraction of argan oil occurred at the Adrar station of the National Institute of Forestry Research of Algeria. Fruits from native and introduced *A. spinosa* were first sun-dried for 14 days and peeled to extract the nuts. Then, the nuts were cracked, and the kernels were extracted by the mechanical screw press Komet DD 85 G (IBG Monforts Oekotec GmbH & Co. KG, Mönchengladbach, Germany) at <45 °C for ~10 min. This mechanical press operates using standardized manufacturer parameters for cold pressing. The extruder’s rotational speed is set at 85 rpm and the pressure applied is preset by the hydraulic system. This cannot be adjusted by the operator and remains constant throughout the process. The obtained oil samples were decanted, filtered and their yield was gravimetrically measured. Overall, a total of n = 3 samples of oil coming from the natural argan grove (Tindouf) and n = 3 samples of argan oil coming from the argan plantation (Adrar) were produced and stored at 4 °C in hermetically sealed, dark, and sterile glass bottles, where n = 3 indicates three technical replicates. To prevent natural oxidation phenomena, all analyses were conducted within one month of the oils’ production.

### 2.3. Reagents and Materials

Organic solvents (i.e., n-heptane, n-hexane, diethyl ether, and methanol) were reagent-grade and provided by J.T. Baker (Phillipsburg, NJ, USA). Other organic solvents (i.e., ethyl acetate, methanol, and water) were HPLC-grade and purchased from LiChrosolv (Merk, Darmstadt, Germany). Inorganic reagents [i.e., H_2_O_2_ (30%, *v*/*v*) and HNO_3_ (65%, *v*/*v*)] and ultrapure water were supplied by J.T. Baker (Milan, Italy). Bis-trimethylsilyl-trifluoroacetamide and trimethylchlorosilane (BSTFA:TMCS, 99:1) and silica gel type G, with ~13% CaSO_4_ (high-purity grade), were provided by Supelco (Bellefonte, PA, USA). The Folin–Ciocalteu reagent was obtained from Sigma-Aldrich (Steinheim, Germany). Commercial standards of fatty acid methyl esters (FAMEs, C_4_–C_24_), single sterols (cholesterol, stigmasta-8,22-dien-3β-ol, campesterol, stigmasterol, spinasterol, stigmasta-7,24-dien-3-ol, schottenol and ∆-7-avenasterol, ≥98% purity each), single tocopherols (α-tocopherol, β-tocopherol, γ-tocopherol, δ-tocopherol, 98% purity each), gallic acid (99% purity), and squalene (≥98%purity) were manufactured by Sigma-Aldrich (St. Louis, MO, USA) and Supelco (Bellefonte, PA, USA). Reference standards of Na, Mg, K, Ca, Fe, Cu, Mn, Zn, Co, Se, Cr, Ni, As, Cd, and Pb (1000 mg/L in 2% HNO_3_, each) were bought from Fluka (Milan, Italy). Depending on the targeted analyte, the following internal standards were employed: tetradecane (99% purity, Merck Life Science S.r.l., Milan, Italy), α-cholestanol (TraceCERT^®^ grade, Supelco, Bellefonte, PA, USA), and rhenium (Re, 1000 µg/mL in 5% HNO_3_, LGCStandards, Teddington, UK).

### 2.4. Analysis of Physicochemical Properties

Physicochemical parameters of argan oils were studied by employing protocols relative to vegetable oils derived from standard and official methods of analysis. The refractive index (RI) of oil samples was measured by an Abbe refractometer (Model RMT, Optech, Milano, Italy), according to the NF T60-212 standard method [22]. The saponification number was assessed according to the AOAC Official Method 920.160 [23]. Briefly, an oil sample was refluxed with 25 mL of methanolic KOH, followed by back-titration of the remaining KOH with HCl and using phenolphthalein as an indicator. A blank titration was conducted in parallel. The saponification index was determined by the formula:

$$\mathrm{Saponification}\;\mathrm{index}\;(\mathrm{mgKOH}/g)\;=\frac{\left(V0-V1\right)\times N\times KOHmw}{w}$$

where V0 is the titrant volume (ml of HCL) used for the blank titration, V1 is the titrant volume (ml of HCl) used for the test titration, N is the HCl normality (0.5), KOH_MW_ is the molecular weight of KOH (56.1 g/mol), and *w* is the weight of the oil sample (2.0 g).

The acidity of the oil was assessed by dissolving the oil sample in 90 mL of a neutralized ethyl alcohol/diethyl ether solution (1:2, *v*/*v*) containing 1% phenolphthalein as an indicator. The mixture was titrated with KOH until a color change occurred. The acidity was calculated as % of oleic acid using the formula [24]:

$$\mathrm{Oleic}\;\mathrm{acid}\;(\%)\;=\;\frac{N\times V\times AOmw}{w\times 10}$$

where V is the volume of the titrant (ml of KOH), N is the normality of KOH (0.1), OA_MW_ is the molecular weight of the oleic acid (282 g/mol), and *w* is the weight of the oil sample (5.0 g).

The peroxide value was measured by mixing the oil sample with a glacial acetic acid/chloroform solution (3:2, *v*/*v*, 25 mL) and a saturated KI solution (500 μL). After shaking and keeping the mixture in the dark (5 min), distilled water (75 mL) and starch indicator were added. The released iodine was titrated with sodium thiosulfate until a color change was observed. The peroxide value was calculated and expressed as milliequivalents of reactive oxygen per kilogram of oil (mEqO_2_/kg) according to the equation [25]:

$$\mathrm{Peroxide}\;\mathrm{value}\;({\mathrm{mEqO}}_{2}/\mathrm{kg})\;=\frac{V\times N\times 1000}{w}$$

where V is the titrant volume (ml of Na_2_S_2_O_3_), N is the normality of the Na_2_S_2_O_3_ solution (0.01), and w is the weight of the oil sample (2.0 g).

An UV–VIS spectrophotometer (UV-2401 PC, Shimadzu, Milan, Italy) was employed for the spectral measurements of argan oils. To this purpose, the absorption coefficients K270 (at 270 nm) and K232 (at 232 nm) were determined according to the methodology described in the standard of the Commission Regulation (EEC) No. 2568/91 [26].

### 2.5. Fatty Acid (FA) Composition

FA composition was determined following the protocol of Lo Turco et al. already employed for cold-pressed oils [27]. Briefly, every oil sample (0.1 g) was transesterified with n-heptane (2 mL) and methanolic KOH (0.2 mL) to obtain fatty acid methyl esters (FAMEs) and, subsequently, decanted. The upper layer was collected and analyzed by gas chromatography coupled with a split/splitless injector and flame ionization detector (GC-FID, Dani Master GC1000, Dani Instrument, Milan, Italy). To this purpose, a SLB-IL100 capillary column (Supelco, Sigma Aldrich, Burlington, MA, USA) was used and the oven temperature ranged from 165 to 210 °C at a rate of 2 °C/min, and was then held at the maximum temperature for 10 min. Helium was used as the carrier gas (linear velocity of 30 cm/s), along with an injection volume of 1 µL and a split ratio of 1:100. FAMEs of nutritional interest were identified by comparison with reference standards and quantified using the percentage peak area method.

### 2.6. Phytosterol Composition

The sterol profile of wild and domesticated oils was determined in accordance with the EU Regulation No. 1348/2013 on the standardization of the physical, chemical, and analysis methods for olive oil within the European Union [28]. Every oil sample was spiked with α-cholestanol as an internal standard and saponified using ethanolic KOH. The unsaponifiable fraction was loaded on activated silica gel plates to conduct the separation of sterols by thin-layer chromatography (TLC). Elution was carried out in 45 min with 100 mL of n-hexane/ethyl ether (65:35 *v*/*v*) by using a developing chamber (27.0 × 26.5 × 7.0 cm), and sterol bands were visualized under UV light with 2,7-dichlorofluorescein. The sterol fraction was scraped off the silica gel, extracted with hot ethyl acetate, and the solvent removed under vacuum. The residue was derivatized with BSTFA–TMCS to obtain trimethylsilyl ether derivatives, which were analyzed by GC/FID using a SPB-1 capillary column (Supelco, Bellefonte, PA, USA) as already described in detail by Slimani and colleagues [18]. Sterols were identified by comparison with commercial standards and quantified using both the percentage peak area method (results expressed as %) and the internal standard method (results expressed as mg/100 g).

### 2.7. Determination of Tocopherols

The protocol already reported by Amar et al. [29] for carob oil served to determine α-, β-, γ-, and δ-tocopherols. Briefly, every oil sample was (0.1 g) diluted in n-hexane, filtered, and directly analyzed by high-performance liquid chromatography with fluorescence detection (HPLC-FD, Shimadzu, Milan, Italy). Separation was performed on a LiChrosorb^®^ Si60 column protected by a LiChroCART 4–4 guard column (Merck KgaA, Darmstadt, Germany), using n-hexane/ethyl acetate (90:10, *v*/*v*) under isocratic conditions at 40 °C and at 8 mL/min, and an injection volume of 20 µL. Tocopherols were identified by comparison with commercial standards (295 nm excitation, 330 nm emission) and quantified by using both the percentage peak area method (results expressed as %) and the external standardization procedure (results expressed as mg/kg).

### 2.8. Squalene Analysis

The levels of squalene in argan oils were assessed by following the method proposed by Cicero et al. for other cold-pressed oils [30]. The oil sample (0.1 g) was spiked with the internal standard tetradecane and purified by silica solid-phase extraction (SPE) with n-hexane. Analysis was performed by gas chromatography coupled with mass spectrometry (GC-MS, Shimadzu GC-2010/QP-2010 Plus, Shimadzu, Milan, Italy) using an SPB-5MS capillary column (Supelco, Bellefonte, PA, USA). The oven temperature was programmed from 80 °C (held for 1 min) to 140 °C at 20 °C/min and, finally, to 290 °C (held for 2 min) at 5 °C/min. Injection volume was 1 µL, with a 1:10 split ratio. For the MS parameters, the EI source temperature was at 230 °C, the ionization energy and emission current were respectively set at 70 eV and 250 µA, while the interface temperature was equal to 290 °C. Identification was carried out in full-scan mode (mass range: 40–400 *m*/*z*) by comparing the retention time and mass spectra of the analyte with those of the commercial standard. Quantification was performed in selected ion monitoring (SIM) mode, using characteristic fragments (121, 137, 161, and 175 *m*/*z*) and according to the internal standardization.

### 2.9. Total Polyphenol and Carotene Assays

Total polyphenols in argan oils were determined by following the procedure described by Albergamo et al. [31]. Shortly, the oil sample (2.0 mL) was extracted with a solution of methanol/acetic acid/water (50:8:42, *v*/*v*/*v*), stirred for 20 min, and centrifuged (5 min at 6630× *g*). The supernatant was filtered and mixed with the Folin–Ciocalteu reagent (5 mL) and a 20% Na_2_CO_3_ solution (10 mL). After 2 h incubation in the dark, the absorbance of the solution was measured at 760 nm using a UV–Vis spectrophotometer (Shimadzu UV-2401 PC, Shimadzu, Kyoto, Japan). The quantification of total phenols was performed using a calibration curve of gallic acid, and results were expressed as mg gallic acid equivalents per kg of oil (mg GAE/kg). Total carotene content was evaluated by following the protocol employed by Blasi and colleagues for hempseed oil [32]. In brief, every oil sample (1.0 g) was added with diethyl ether (50 mL), vortexed, and sonicated for 1 min. The absorbance of the mixture was measured by an UV-Vis spectrophotometer (UV-2401 PC, Shimadzu, Milan, Italy) at 663 nm (A663) and 640 nm (A640), for chlorophyll A (Chl-A) and chlorophyll B (Chl-B), respectively, while the total carotene content was determined at 470 nm (A470). Hence, the concentration (μg/mL) of these pigments was calculated by the formulas of Izzo et al. [33]:

Chl-A = 9.93 × A663 − 0.78 × A640

Chl-B = 17.60 × A640 − 2.81 × A663

Chl-A + Chl-B = 7.12 × A663 − 16.80 × A640

Total Carotene = (1000 × A470 − 0.52 × Chl-A − 7.25 × Chl-B)/226

### 2.10. Determination of Inorganic Elements

Argan oils were extracted and analyzed for inorganic elements according to our previous work on certain Tunisian edible oils [34]. Specifically, the oil sample (0.3 g) was spiked with Re as an internal standard and mineralized with 8 mL of HNO_3_ and 2 mL of H_2_O_2_ by a microwave digestion system (Ethos I, Milestone Spa, Bergamo, Italy). The digests were subsequently diluted to 25 mL with distilled water and filtered. The elemental determination was performed by a quadrupole ICP-MS (iCAP Q, Thermo Scientific, Waltham, MA, USA). The operating conditions were as follows: incident RF power equal to 1550 W, and plasma (Ar), auxiliary (Ar), and carrier (Ar) gases at flow rates of 14 L/min, 0.8 L/min, and 1.10 L/min, respectively. The instrument was operated in He collision mode (4.7 mL/min) with the spray chamber set at +2.7 °C. The injection volume and sample introduction rate were 200 µL and 0.93 mL/min, respectively. The spectra were acquired in full scan mode, with dwell times of 0.5 or 0.01 s/point, depending on the analyte. The quantification procedure occurred by internal standardization.

### 2.11. Statistical Analysis

For every type of argan oil, the experimental data were expressed as mean ± standard deviation of n = 3 oil samples, where every sample was analyzed in triplicate. Statistical analyses were conducted by R Studio v. 4.2.1. First, the normal distribution of data was verified by a Shapiro–Wilk test, and then a two-tailed Student’s *t*-test for unpaired data was applied to determine if the means of two independent sample groups (i.e., oil from plantation and oil from natural forest) were significantly different. Statistical significance was at *p* ≤ 0.05.

## 3. Results and Discussions

### 3.1. Yield and Physicochemical Traits of Argan Oils

Table 2 presents the extraction yield and physicochemical characteristics of experimental argan oils.

Non-significantly different extraction yields (i.e., 38.63% vs. 39.89%, *p* > 0.05) and refractive indices (1.469 vs. 1.470, *p* > 0.05) were obtained for both types of oils. The literature from the last decade reports variable yields of the argan oil, ranging from 16% to 66%. Most of these studies linked the yield variability to the extraction process, namely drying of fruits, the eventual roasting of kernels, and the type of extraction of seeds as well (i.e., mechanical press, traditional, solvent or supercritical fluid extraction) [13,36,37,38,39], and just a few studies examined the extraction yield in relation to other influential factors, such as genotype and geographical origin of *A. spinosa* [40,41,42].

However, significant differences were found for the main physicochemical properties. In fact, the oil from natural forest showed a higher saponification number (193.11 mg KOH/g vs. 189.69 mg KOH/g, *p* < 0.05), a lower acidity (0.45% vs. 0.63%, *p* < 0.05) and peroxide value (5.00 mEqO_2_/kg vs. 8.88 mEqO_2_/kg, *p* < 0.05) than the oil from plantation. Considering the UV exam, only the K232 coefficient was significantly different between the oil samples from Tindouf and Adrar (respectively 1.46 vs. 1.66, *p* < 0.05), while the other extinction coefficients were similar and non-significantly different (*p* > 0.05). Recently, two studies assessed the impact of cultivation of *A. spinosa* outside its natural habitat on the nutritional quality of the oil in the northeast and southwest of Morocco [43,44]. Both studies highlighted that the Moroccan argan oil from native and introduced trees was characterized by free acidity in the range of 0.40–0.64% and 0.15–0.55% respectively, and peroxides between 0 and 2.17 mEqO_2_/kg and 0 and 3.30 mEqO_2_/kg, respectively, while the K232 varied between 0.88 and 1.70 and 0.88 and 1.37, and K270 was equal to 0.12–0.34 and 0.09–0.21 respectively [43,44]. However, it is well established that these parameters do not reflect the intrinsic quality of the oilseed source; in contrast, they are typically affected by the post-harvest seed management, poor handling, improper storage, or the age of the oil.

Currently, Algeria does not have official guidelines for the classification of argan oil. Consequently, the country and the international scientific community as well use the quality standards on argan oil set in 2003 by the Moroccan Institute for Normalization as a reference framework, since they are the most widely recognized and extensively applied criteria for the evaluation of argan oil quality and authenticity [35].

According to these standards, the refractive index should vary from 1.463 to 1.472 at 20 °C, while the saponification value should be in the range 189.0–199.1 mgKOH/g. The same guidelines classify the argan oil into four grades based on the acidity, peroxide value and UV assay: extra-virgin (acidity: <0.8%, peroxides: ≤15 mEqO_2_/kg, and K270: ≤0.35), virgin (acidity: <1.5%, peroxides: ≤20 mEqO_2_/kg, and K270: ≤0.35), pure (acidity: <2.5%, peroxides: ≤20 mEqO_2_/kg, and K270: ≤0.35) and lampante (acidity: >2.5%) [35,45]. The Moroccan standards have not yet established a limit for the K232. Nevertheless, Kharbach et al. suggested that this absorption coefficient should not exceed 2.52 for extra-virgin argan oil [46].

Despite the slight differences in chemical–physical properties, the argan oils from native and introduced *A. spinosa* trees are both high-quality oils, since they can be considered extra-virgin oils according to the Moroccan guidelines (Table 2).

### 3.2. FA Composition

Table 3 reports the FA composition of investigated argan oils. Perfectly comparable data were obtained for the argan oil from Tindouf and Adrar, since the statistical analysis did not reveal any statistically significantly different values for the FAs measured. This evidence suggests that the plantation of *A. spinosa* in new Algerian areas may not significantly impact the quality of the FA profile of the argan oil.

Overall, monounsaturated FAs were the most abundant FAs (MUFA, 48.33–47.15%, *p* > 0.05), followed by polyunsaturated FAs (PUFA, 30.92–31.43%, *p* > 0.05) and saturated FAs (SFA, 20.18–20.69%, *p* > 0.05). Oleic acid and linoleic acid accounted for almost all the MUFA (C18:1n-9, 46.63–45.48%, *p* > 0.05) and PUFA (C18:2n-6, 30.84–31.35%, *p* > 0.05), respectively. Considering SFA, palmitic and stearic acids were the most abundant FAs (C16:0, 13.82–14.11%, and C18:0, 5.69–5.87%, *p* > 0.05). Interestingly, cis-vaccenic acid, a n-7 MUFA, was revealed in significant amounts in both argan oils (C18:1n-7, 1.10–1.11%, *p* > 0.05). This FA is already known to be naturally present in many oilseeds (e.g., rapeseed, sunflower, soybean, flaxseed, pumpkin and sesame) in percentages between 0.4 and 2.12% [47,48] and, recently, was also reported in Moroccan argan oil (0.39–0.40%) [49]. Interestingly, cis-vaccenic acid showed anti-obesity mechanisms in mammals, including reduced food intake, decreased lipid accumulation, and regulation of genes involved in fatty acid oxidation and lipogenesis [50].

Overall, the FA composition of both argan oils covered the percent ranges expected for extra-virgin argan oil by the Moroccan guidelines (Table 3) [35,45].

The literature from the last ten years has confirmed that the argan oil is a valuable source of nutritionally relevant MUFA and PUFA and that the predominant FAs are oleic acid (41.14–58.70%), followed by linoleic and palmitic acids (respectively 22.30–36.95% and 11.80–18.69%), and, finally, stearic acid (3.00–7.59%) [36,43,44,46,49,51,52,53,54]. Overall, results from our study fall within the quantitative ranges already described for Moroccan, Algerian and Spanish oils.

Nonetheless, differently from our findings, recent studies demonstrated that the FA composition of argan oil from plantation varied from that from natural groves, especially with respect to the most abundant FAs [43,44]. However, available data suggest that the variability of the FA composition across these studies’ scenarios could be linked to the interplay between genetic and ripeness characteristics of the argan fruit, on the one hand, and the environmental conditions of the areas where trees grew, on the other, rather than to the cultivation of *A. spinosa* itself [43,44,55,56].

### 3.3. Phytosterol Composition

Table 4 shows the sterol composition (%) and total sterols (mg/100 g) of investigated argan oils. Similar profiles were outlined for investigated oils. In fact, oils from natural and planted trees were characterized by the highest percentages of spinasterol (38.89% vs. 36.06%, *p* > 0.05) and schottenol (47.93% vs. 46.67%, *p* > 0.05), followed by Δ7-avenasterol (5.41% vs. 6.82%, *p* < 0.05), stigmasta-7,24-dien-3-ol (3.84% vs. 5.13%, *p* < 0.05) and stigmasta-8,22-dien-3β-ol (3.55% vs. 3.60%, *p* > 0.05). Other compounds present at lower and non-significantly different levels in both oils were cholesterol, campesterol, and stigmasterol (<0.4%, *p* > 0.05). Coherently, respective total sterols amounted to 143.26 mg/100 g and 140.97 mg/100 g, with a non-significant difference between oils from Tindouf and Adrar (*p* > 0.05, Table 4).

As for the other parameters discussed so far, the sterol profile confirmed that both argan oils were of high quality, since the single congeners complied with the Moroccan limits established for extra-virgin argan oil [35]. Moreover, the plantation of *A. spinosa* outside its biotope did not affect the sterol composition of the oil. This conclusion was also reached by Sabiri and colleagues, who compared phytosterols of argan oil from natural forest stands and from an argan tree plantation in Southwestern Morocco [43].

A literature review also confirmed that the sterol profile of studied oils was in line with those reported by other studies. In fact, total sterols of Moroccan argan oils typically amounted to 140.5–233.0 mg/100 g, and schottenol, spinasterol and Δ7-avenasterolwere the most representative compounds, with variable amounts between 33.22 and 45.40%, 27.49 and 41.2%, and 3.07 and 4.5%, respectively [43,49,52,53].

Notoriously, phytosterols are intended not only as bioactives with beneficial health impacts but also as useful markers of oil authentication and adulteration, being considered the “fingerprint” of a vegetable oil [57,58]. In this respect, the most abundant species of argan oil, namely spinasterol and schottenol, are known to act as potent antioxidants, anti-inflammatory agents, and potential neuroprotective compounds, offering a significant potential in preventing age-related diseases [59]. On the other hand, campesterol, which must be present in argan oil at very low levels (≤0.4% [35]), has already been considered as a marker for testing oil authenticity [60].

### 3.4. Tocopherols

Single tocopherols (expressed as % and mg/kg) and total tocopherols (mg/kg) of argan oils from this study are reported in Table 5.

Overall, the isomers were found in the following decreasing order: γ > δ > α > β. Coherently, γ-tocopherol was the most concentrated species (up to 746.78 mg/kg) and accounted for up to 88.63% of total tocopherols, while β-tocopherol was the least abundant one (up to 2.32 mg/kg) and accounted for up to 0.28% of investigated compounds. Most tocopherols were significantly different between samples under study. Specifically, α-and γ-isoforms were significantly higher in the argan oil from the forest stand (*p* < 0.05), while δ-tocopherol was higher in the oil from cultivated trees (*p* < 0.05). However, when the concentrations were converted from mg/kg to percentage values, the relative variance became proportionally smaller, making the comparisons non-statistically significant (*p* > 0.05, Table 5). Finally, the total tocopherol content was found to be significantly higher in the oil from endemic *A. spinosa* trees than in that from planted trees (842.61 mg/kg vs. 627.63 mg/kg). Overall, the tocopherol profile of both argan oils investigated in this study was in accordance with the parameters established for tocopherols by the Moroccan guidelines for extra-virgin argan oil (Table 5).

Similar findings to those from our study were reached by Azizi and colleagues, who studied the tocopherol profile of Moroccan argan oil from trees introduced in Oujda City and from trees in their natural biotope [44]. Indeed, total tocopherols of oil from the argan grove (417.50–553 mg/kg) were higher than total tocopherols of oil obtained from planted trees (323.86–527.24 mg/kg), due to higher levels of α-tocopherol (36.34–63.70 mg/kg vs. 25.72–48.34 mg/kg) and γ-tocopherol (341.61–465 mg/kg vs. 247.45–447.66 mg/kg).

However, no clear trend could be identified in the tocopherol profiles of Moroccan oils from two natural forest stands in the regions of Essaouira and Taroudant and from a plantation in Casablanca [43], since total tocopherols were more abundant in argan oils from Casablanca and Essaouira and lower in the oil from Taroudant. Furthermore, this trend was determined solely by γ-tocopherol, which accounted for nearly all the compounds analyzed (up to 90.53%).

In general terms, the different contents of single and total tocopherols found in argan oils obtained from *A. spinosa* inside and outside its natural biotope could be related to the different pedoclimatic contexts where the trees grew (see Table 1), especially in terms of distance from the coast and altitude [61]. However, the metabolism of tocopherols in plants is regulated not only by environmental factors but also by several biosynthetic pathways, enzymatic activities, and genetic factors [62]. For instance, when subjected to stressful conditions, such as drought, excessive light exposure or high temperatures, plants can boost tocopherol production as a defense mechanism against oxidative damage [62]. Consequently, the hyper-arid environment of the Adrar province may have influenced the tocopherol metabolism of the tree, particularly in terms of increased synthesis of δ-tocopherol in the fruit and, consequently, in the oil derived.

A review of compositional studies on argan oil conducted over the past decade has highlighted that α-, γ- and δ-isoforms were in the range 4.24–7.04%, 87.15–90.53% and 4.04–7.64% respectively, while β-tocopherol was between not detected-0.12% [43,52,53]. While acknowledging the considerable variability inherent in these ranges, our results were nonetheless consistent with these %values.

### 3.5. Squalene, Total Polyphenols and Carotenes

The Algerian argan oils were also investigated for minor compounds such as squalene, total polyphenols and carotenoids. Overall, the introduction of *A. spinosa* trees in a new biotope affected the levels of such phytochemicals, since they were significantly higher in the oil produced in Tindouf than in that from Adrar. Specifically, squalene levels were equal to 2537.33 mg/kg in the oil from the natural forest and 2144.33 mg/kg in the oil obtained from the argan plantation (*p* < 0.05). Similarly, the oil extracted from wild argan nuts was characterized by higher total polyphenols (60.29 vs. 45.20 mg/kg, *p* < 0.05) and carotenes (19.30 vs. 15.86 mg/kg, *p* < 0.05) (Table 6).

Differently from the other nutrients discussed so far, no guideline values have been established for these compounds that can be used to determine the quality of the oil, and scarce and dated literature is available for comparative purposes.

Khallouki and coworkers determined levels of squalene in Moroccan argan oils, similarly to our study, since it ranged between 3030 and 3210 mg/kg depending on the commercial quality (i.e., commercial, food and cosmetic) of the oil itself. However, the same study reported that total polyphenols varied between 3.26 and 3.7 mg/kg in these oils, being much lower than our results [63].

In an array of samples of virgin argan oil from Southwestern Morocco, the polyphenol content ranged between 6.07 and 152.04 mg/kg oil [64], while a recent comparative study on the antioxidant activity of olive oil and argan oil reported that the olive oil had higher total phenols than argan oil (i.e., 175.91 mg/kg oil and 36.24 mg/kg, respectively). The same study showed respective contents of total carotenoids equal to 0.34 mg/kg and 0.24 mg/kg, which are much lower than total carotenes assessed in Algerian oils [65]. However, Gharbi and colleagues studied the impact of deodorization on the chemical composition of argan oil, and they spectrophotometrically measured contents of β-carotene before and after deodorization of the oil (i.e., 13.50 mg/kg and 11.5 mg/kg, respectively), which were lower, but still congruent, than total carotenes from this study [52]. Overall, the variability of described data can be explained by the complex influence of genetic, environmental, and technological factors (fruit processing, oil extraction, and storage conditions) on the pattern of bioactives of argan oil.

However, squalene is undoubtedly a key bioactive of the argan oil’s chemical profile, not only for its relevant presence in the oil but also for the several cosmetic and health benefits provided, such as antioxidant, anti-aging, and moisturizing effects [9]. Interestingly, although the argan oil contains lower levels of squalene (approximate range: 2000–3000 mg/kg) than olive oil (approximate range: 2000–7000 mg/kg), its levels are significantly higher than those of many other fruit and seed oils [30,66,67,68].

### 3.6. Inorganic Elements

The element profile of argan oils from *A. spinosa* trees native to Tindouf and introduced in Adrar is reported in Table 7. Considering major elements, they were quantitatively determined in the order: Na > Ca ≈ K > Mg. Specifically, they varied from 64.08–47.83 mg/kg (Na) to 7.65–4.71 mg/kg (Ca). On the other hand, trace elements followed the order: Se > Fe ≈ Zn ≈ Cr ≈ Co > Cu > Mn. Interestingly, Se, the most abundant trace element, amounted to 1.07–0.91 mg/kg in the oils, while Mn, the least concentrated metal, was equal to 0.12–0.03 mg/kg. Among toxic and potentially toxic elements, the most abundant metal was Ni (0.65–0.41 mg/kg), while heavy metals, such as As, Cd and Pb, were present at lower levels (0.01–0.04 mg/kg) (Table 7).

Every investigated element was more concentrated in the oil from planted specimens than in that from endemic trees, with a statistical significance at *p* < 0.05 (Table 7). Hence, it can be stated that the cultivation of *A. spinosa* outside its natural area significantly affected the profile of organic elements. Since the argan oils from this study were obtained from selected ripe fruits that underwent the same type of processing and extraction, the significant variations in elemental concentrations were primarily due to the different geographical origins. In fact, the growth context of *A. spinosa*, with its variables (see Table 1), defines the pool of inorganic elements in the soil, which, in turn, are adsorbed by the tree and fruits [73]. Moreover, another factor potentially responsible for the difference between the described element profiles could be the agronomic management of planted trees: it did not involve any chemical inputs, but did include the drip irrigation, which, in turn, affects the mineral content of the soil [74].

The literature on the element profile of the argan oil is limited and quite fragmented, since previous studies focused only on macroelements and/or some trace elements, with very little attention paid to toxic and potentially toxic trace elements [69,70,71,72]. Overall, wide quantitative ranges could be established for these analytes, due to the many factors influencing the concentration of inorganic elements in any vegetable oil [69,70,71,72]. However, as can be easily seen from Table 7, except for certain elements (e.g., Na and Zn), our results proved to be generally in line with literature data.

### 3.7. Limitations of the Study

This is the first study conducted in Algeria to comprehensively compare the quality of argan oil derived from natural populations and from trees cultivated outside their native habitat. However, certain limitations must be acknowledged to ensure that the experimental evidence is properly interpreted.

Firstly, the comparison was conducted in only two geographical locations that differ in terms of both the growth status of the trees (wild vs. planted) and soil and climate conditions. Consequently, it is not possible to fully separate the effects of domestication and the environment, and conclusions regarding the impact of tree cultivation on the overall quality of the derived oil should be treated with caution. Future studies will address this limitation by including a larger number of sites and conducting a more in-depth analysis of various influencing environmental factors over a longer study period, for example.

Secondly, the small sample size (three analytical replicates per group) limits the statistical robustness and prevents the robust application of supervised multivariate models. In this study, the statistical analysis was therefore based on pairwise comparisons using *t*-tests, which are suitable for identifying specific differences, but not for capturing complex multivariate patterns. Hence, approaches such as principal component analysis (PCA), hierarchical cluster analysis (HCA) and partial least squares discriminant analysis (PLS-DA) requiring larger datasets should be implemented in future multivariate analyses, not only to ensure statistical robustness and avoid overfitting but also to further strengthen the discrimination and interpretation of sample variability.

Despite these limitations, the present study provides, for the first time, a comprehensive characterization of argan oil produced in Algeria, surpassing many studies conducted on Moroccan oil. Hence, this study constitutes a solid scientific basis for evaluating, in the future, the quality of oil obtained from *A. spinosa* cultivated in new agroecological contexts more exhaustively.

## 4. Conclusions

This study provides the first comprehensive comparison of argan oil obtained from natural populations of Argania spinosa in Tindouf and from trees cultivated outside their native habitat in Adrar, Algeria. The results showed that both oils exhibited physicochemical characteristics, fatty acid composition, and sterol profiles consistent with the quality requirements established by the Moroccan guidelines for extra-virgin argan oil. In addition, several nutritional attributes, particularly fatty acid and phytosterol composition, were found to be broadly comparable between the two oil types.

At the same time, significant differences were observed in the concentrations of tocopherols, squalene, total polyphenols, carotenoids, and inorganic elements, which generally occurred at higher levels in the oil obtained from natural populations. These differences likely reflect the influence of distinct pedoclimatic and agronomic conditions, although the relative contribution of environmental and cultivation-related factors could not be fully distinguished in the present study.

Overall, the findings suggest that argan oil produced from cultivated *A. spinosa* trees in Adrar can achieve quality characteristics comparable to those of oil obtained from native populations for several key compositional parameters. However, the present work is limited to the characterization of oil quality and composition. Therefore, the results should not be interpreted as definitive evidence of the long-term agronomic suitability, ecological adaptability, or large-scale cultivation feasibility of *A. spinosa* outside its natural biotope.

Nevertheless, this study provides valuable baseline data on the quality of Algerian argan oil and contributes to ongoing efforts aimed at the conservation, valorization, and sustainable development of this species in Algeria.

## Figures and Tables

**Table 1:** Geographical coordinates and geopedoclimatic characteristics of the Algerian provinces of Tindouf and Adrar involved in the study.

Sampling Site	Geographic Coordinates	Distance from the Sea	Altitude	Annual Precipitation	Annual Temperature	Soil
Tindouf	28.459865° N 8.147605° W	165 Km	530 m	60.3 mm	25 ± 7.16 °C	Sandy
Adrar	27.87718° N 0.27909° W	951 Km	257 m	33.5 mm	24 ± 8.1 °C	Sandy clay

**Table 2:** Extraction yield and physicochemical properties of argan oil from natural (Tindouf) and planted (Adrar) *A. spinosa* trees. Results are expressed as mean ± standard deviation of n = 3 samples per oil type, each sample being analyzed three times. The table also shows the coefficient of variation (%CV) calculated for the parameters of both types of oil and the Moroccan guideline values for extra-virgin argan oil [35].

Parameter	Oil from Natural Forest (Tindouf)	Coefficient of Variation (%CV)	Oil from Plantation (Adrar)	Coefficient of Variation (%CV)	Moroccan Guidelines for Extra-Virgin Oil [35]
Yield (%)	38.63 ± 1.85	4.8	39.89 ± 0.56	1.4	-
Refractive index (at 20 °C)	1.469 ± 0.00	-	1.47± 0.00	-	1.463–1.472
Saponification number (mg KOH/g oil)	193.11 ± 2.12 *	1.1	189.69± 3.06 *	1.6	189.0–199.1
Free acidity (%)	0.45 ± 0.03 *	6.7	0.63 ± 0.07 *	11.1	<0.8
Peroxide value (mEqO_2_/kg)	5.00 ± 0.34 *	6.8	8.88 ± 0.52 *	5.9	≤15
K232	1.46 ± 0.04 *	2.7	1.66 ± 0.02 *	1.2	-
K270	0.20 ± 0.02	10	0.25 ± 0.03	12	≤0.35
ΔK	0.001 ± 0.001	100	0.002 ± 0.001	50	-

* Significantly different values at *p* < 0.05.

**Table 3:** FA composition (%) of argan oil from natural (Tindouf) and cultivated (Adrar) *A. spinosa* trees. Results are expressed as mean ± standard deviation of n = 3 samples per oil type, each sample being analyzed three times. The table also shows the Moroccan guideline values for extra-virgin argan oil [35].

FAs	Oil from Natural Forest (Tindouf)	Oil from Plantation (Adrar)	Moroccan Guidelines for Extra-Virgin Oil [35]
C12:0	0.04 ± 0.01	0.05 ± 0.01	-
C14:0	0.11 ± 0.01	0.12 ± 0.01	≤0.2
C16:0	13.82 ± 1.16	14.11 ± 0.83	11.5–15.0
C17:0	0.07 ± 0.01	0.08 ± 0.01	traces
C18:0	5.69 ± 0.54	5.87 ± 0.53	4.3–7.2
C20:0	0.40 ± 0.03	0.39 ± 0.03	≤0.5
C22:0	0.03 ± 0.01	0.05 ± 0.01	≤0.2
C24:0	0.02 ± 0.01	0.03 ± 0.01	-
SFA	20.18 ± 1.66	20.69 ± 1.31	*-*
C16:1 n-9	0.02 ± 0.01	0.02 ± 0.00	-
C16:1 n-7	0.17 ± 0.01	0.15 ± 0.01	≤0.2
C17:1	0.03 ± 0.01	0.02 ± 0.01	-
C18:1 n-9	46.63 ± 2.46	45.48 ± 2.71	43.0–49.1
C18:1 n-7	1.11 ± 0.14	1.10 ± 0.13	-
C20:1 n-9	0.37 ± 0.03	0.38 ± 0.02	-
MUFA	48.33 ± 2.43	47.15 ± 2.83	-
C18:2 n-6	30.84 ± 0.90	31.35 ± 1.71	29.3–36.0
C18:3 n-6	0.02 ± 0.01	0.02 ± 0.00	-
C18:3 n-3	0.06 ± 0.01	0.06 ± 0.01	≤0.3
PUFA	30.92 ± 0.90	31.43 ± 1.70	-

**Table 4:** Sterol composition (%) and total sterols (mg/100 g) of argan oil from native (Tindouf) and planted (Adrar) *A. spinosa* trees. Results are expressed as mean ± standard deviation of n = 3 samples per oil type, each sample being analyzed three times. The table also shows the Moroccan guideline values for extra-virgin argan oil [35].

Phytosterol	Oil from Natural Forest (Tindouf)	Oil from Plantation (Adrar)	Moroccan Guidelines for Extra-Virgin Oil [35]
Cholesterol	0.38 ± 0.03	0.35 ± 0.02	≤0.4
Campesterol	0.16 ± 0.01	0.14 ± 0.02	≤0.4
Stigmasterol	0.27 ± 0.03	0.24 ± 0.01	-
Stigmasta-8,22-dien-3β-ol	3.55 ± 0.22	3.60 ± 0.34	3.2–5.7
Spinasterol	38.89 ± 1.84	36.06 ± 1.65	34.0–44.0
Stigmasta-7,24-dien-3-ol	3.84 ± 0.12 *	5.13 ± 0.10 *	-
Schottenol	47.93 ± 2.35	46.67 ± 2.20	44.0–49.0
Δ7-avenasterol	5.41 ± 0.13 *	6.82 ± 0.24 *	4.0–7.0
Total sterols	143.26 ± 7.56	140.97 ± 4.57	≤220

* Significantly different values at *p* < 0.05.

**Table 5:** Single tocopherols (% and mg/kg) and total tocopherol content (mg/kg) of argan oil from native (Tindouf) and planted (Adrar) *A. spinosa* trees. Results are expressed as mean ± standard deviation of n = 3 samples per oil type, each sample being analyzed three times. The table also shows the Moroccan guideline values for extra-virgin argan oil [35].

Tocopherol	Oil from Natural Forest (Tindouf)		Oil from Plantation (Adrar)		Moroccan Guidelines for Extra-Virgin Oil [35]	
	mg/kg	%	mg/kg	%	mg/kg	%
α-tocopherol	36.04 ± 2.51 *	4.28 ± 0.42	32.23 ± 2.10 *	5.14 ± 0.75	-	2.4–6.5
β-tocopherol	2.32 ± 0.67	0.28 ± 0.08	1.25 ± 0.95	0.20 ± 0.08	-	0.1–0.3
γ-tocopherol	746.78 ± 24.14 *	88.63 ± 2.86	529.25 ± 19.33 *	84.33 ± 3.08	-	81.0–92.0
δ-tocopherol	51.22 ± 9.10 *	7.08 ± 1.08 ^†^	64.90 ± 7.81 *	10.34 ± 1.24 ^†^	-	6.2–12.8
Total tocopherols	842.61 ± 28.31 *	-	627.63 ± 21.10 *	-	600–900	-

* Significantly different concentrations (mg/kg) at *p* < 0.05; ^†^ significantly different % values at *p* < 0.05.

**Table 6:** Squalene (mg/kg), total polyphenols (mg GAE/kg), and total carotenes (mg BCE/kg) of argan oil from endemic (Tindouf) and cultivated (Adrar) *A. spinosa* trees. Results are expressed as mean ± standard deviation of n = 3 samples per oil type, each sample being analyzed three times.

Analyte	Oil from Natural Forest (Tindouf)	Oil from Plantation (Adrar)
Squalene	2537.33 ± 16.04 *	2144.33 ± 12.10 *
Total polyphenols	60.29 ± 4.97 *	45.20 ± 3.28 *
Total carotenes	19.30 ± 1.33 *	15.86 ± 1.13 *

* Significantly different values at *p* < 0.05.

**Table 7:** Element profile (mg/kg) of argan oil from natural (Tindouf) and cultivated (Adrar) *A. spinosa* trees. Results are expressed as mean ± standard deviation of n = 3 samples per oil type, each sample being analyzed three times. Comparative literature data are also reported in the table.

Element	Oil from Natural Forest (Tindouf)	Oil from Plantation (Adrar)	Literature Data
Major elements			
Na	64.08 ± 1.59 *	47. 83 ± 2.13 *	162.0–184.10 [69]
Mg	7.65 ± 0.18 *	4.71 ± 0.15 *	0.41–18.00 [69,70,71]
K	13.15 ± 0.28 *	8.03 ± 0.15 *	0.05–11.60 [69,70,71]
Ca	15. 62± 0.52 *	8.25± 0.21 *	6.7–61 [69,70,71]
Trace elements			
Fe	0.59 ± 0.04 *	0.42 ± 0.04 *	0.04–3.00 [69,70,71,72]
Zn	0.58 ± 0.03 *	0.30 ± 0.03 *	0.01–0.23 [69,71]
Cu	0.21 ± 0.02 *	0.13 ± 0.02 *	0.01–0.70 [70,71,72]
Mn	0.12 ± 0.01 *	0.03 ± 0.01 *	0.01–0.07 [70,71,72]
Co	0.42 ± 0.03 *	0.25 ± 0.02 *	0.39–1.14 [69]
Se	1.07 ± 0.05 *	0.91 ± 0.03 *	0.75–1.63 [69]
Cr	0.44 ± 0.03 *	0.24 ± 0.04 *	0.01–0.50 [69,72]
Toxic and potentially toxic trace elements			
Ni	0.65 ± 0.05 *	0.41 ± 0.04 *	3.7–4.0 [69]
As	0.03 ± 0.01 *	0.01 ± 0.00	-
Cd	0.04 ± 0.02 *	0.02± 0.00 *	0.01 [71]
Pb	0.03 ± 0.01 *	0.01 ± 0.00 *	0.01–0.04 [70,72]

* Significantly different values at *p* < 0.05.

## Data Availability

The original contributions presented in this study are included in the article. Further inquiries can be directed to the corresponding author.
